# Long-term increase of fat mass after a four week intervention with fast food based hyper-alimentation and limitation of physical activity

**DOI:** 10.1186/1743-7075-7-68

**Published:** 2010-08-25

**Authors:** Åsa Ernersson, Fredrik H Nystrom, Torbjörn Lindström

**Affiliations:** 1 Department of Medical and Health Sciences, Faculty of Health Sciences, Linköping University, SE 58185 Linköping, Sweden

## Abstract

**Background:**

A sedentary lifestyle and increased consumption of energy dense food have become more common in many parts of the world. The aim of this study was to study long term effects on body composition after a four week intervention with fast food based hyper-alimentation and limited physical activity in young normal weight subjects.

**Methods:**

Eighteen subjects, mean age 26 (6.6) years, increased their energy intake with in average 70% and physical activity were not to exceed 5000 steps/day. Body composition was measured by Dual energy x-ray (DXA) at baseline, after the intervention and after 12 months. A matched control group was also included. ANOVA and Student's paired and unpaired t-test were used.

**Results:**

During the intervention body weight increased with 6.4 (2.8) kg and DXA measurements showed increases of both fat free mass and fat mass. Six months after the intervention the subjects had lost most of the weight gain, - 4.7 (3.1) kg. Twelve months after the intervention body weight had increased with 1.5 (2.4) kg compared to baseline (p = 0.018). DXA measurements at 12 months showed unchanged fat free mass compared to baseline but higher fat mass, + 1.4 (1.9) kg (p = 0.01). After 2.5 years the increase of body weight was 3.1 (4.0) kg (p = 0.01) while there was no change in controls compared to baseline, + 0.1(2.5) kg (p = 0.88).

**Conclusion:**

One year after a short term intervention with increased fast food based hyper-alimentation there was an increase of fat mass but unchanged fat free mass. As the change of fat mass was larger than expected from prospective epidemiological studies and as there was no increase of body weight in controls it raises the issue whether there is a long-term effect to increase fat mass of a short period of hyper-alimentation.

## Background

The prevalence of overweight and obesity has increased in many parts of the world as well as in Sweden during the past few decades [1-3]. Lifestyle factors such as consumption of high energy dense food, but also lack of physical activity, contribute to the development of overweight and obesity [2,4]. Individuals who perform 10 000 steps/day are considered to be physically active while performing less than 5 000 steps/day is defined as having a sedentary lifestyle [5]. In Sweden a 10-year increase of body weight of 3.8 (6.0) kg has been found in individuals aged 25-64 years [6]. Body weight in college students has been found to increase during holidays, although the increase is relatively small, 0.37-0.5 kg [7,8]. Yanovski et al [8] found that weight gain during holidays remained after one year while the college students in the study by Hull et al [9] had returned to pre-holiday body weight when re-examined but that a change of body composition with increase of fat mass, particularly trunk fat mass, and a decrease of fat free mass was found. In a Swedish study on randomly selected individuals aged 37-61 years who underwent DXA measurements, at BMI 25 men were shown to have on average 24% and women 36% body fat [10]. In general, when weight changes occur there are less effects on fat free mass than on fat mass [11]. Fat mass stored in the abdominal region is more associated with health risks than fat mass stored in other regions of the body [2]. Likewise visceral fat mass is more related to metabolic changes and cardiovascular risk than subcutaneous fat mass [12,13]. Women often store body fat in the gluteal-femoral region (gynoid) while men generally have more body fat in the abdominal region (android) [14].

While there is a large number of epidemiological studies on changes of body weight over time in different populations there have not been that many overfeeding experiments to describe effects of increased caloric intake in human beings [15-17] and long-term follow-up after such experiments are rare [18,19]. We prospectively examined effects of rapid weight gain in normal weight individuals. Eighteen healthy young normal weight individuals increased their energy intake and simultaneously their physical activity was not to exceed 5000 steps/day. Mean energy intake during the intervention was 5753 (1495) kcal/day [20]. We have previously reported increased levels of serum ALT and hepatic triglyceride content (HTGC) during the short-term intervention [20]. Further, magnetic resonance imaging showed that men had a larger accumulation of intra abdominal fat volume than women [21]. We also recruited an age and gender matched control group for comparison of anthropometrics.

The aim of this study was to examine long-term changes of body composition after a four week intervention with fast food based hyper-alimentation and limited physical activity in young normal weight subjects and to compare these results with the acute changes of body composition found during the intervention.

## Methods

### Participants and procedure

Eighteen individuals, 12 men and 6 women, with a mean age 26 (6.6) years, increased their energy intake with in average 70%, mainly from fast food, and physical activity were not to exceed 5000 steps/day. Inclusion criteria were being healthy, having normal body weight (BMI < 25) and willingness to increase body weight with 5-15%.

Before starting the hyper-alimentation all participants met a dietician for documentation of their usual eating habits and their individual energy need was calculated based on gender, age and physical activity level. Three-day food records were collected and daily physical activity was 7203 (4104) steps measured with pedometer. Based on the individual calculated energy need the participants were prescribed to double their energy intake during the intervention, by eating at least two fast food based meals per day. The dietary advice was adjusted to fulfil the individually prescribed energy intake and if there was any difficulties to ingest the fast food based diet, it could be changed to any food rich in protein and saturated animal fat the participant accepted with the highest priority to achieve the calculated energy intake. During the study, energy intake was monitored by reports from the subjects and was based on receipts and individual interviews with the participants. All participants in the intervention group carried pedometers before the intervention to get a sense for how much physical activity 5000 steps includes and then during the following weeks they continued to keep low level of physical activity. The intervention has been described in detail elsewhere [20].

The subjects visited the clinic at baseline and every week during the intervention for measurements of body weight and laboratory measurements. These measurements were also performed 6 and 12 months after the intervention. Body composition was measured by Dual energy X-ray Absorptiometry (DXA) at baseline, after 4 weeks and 12 months after the intervention. Body weight was measured 2.5 years after the intervention. Sixteen subjects participated in the 6 month follow-up, the remaining two were abroad studying. Seventeen subjects participated in the 12 month follow-up (1 male subject had become seriously ill, not related to the study, and was excluded from the 12 month follow-up. Further, one male subject had all examinations except body composition determination by DXA).

### Control group

An age and gender matched control group (n = 18), mean age 25 (3.5) years, was recruited and asked not to change their eating habits and physical activity during the next four weeks [20]. They underwent the same anthropometric and laboratory examinations at baseline and after four weeks but not at 6 and 12 months. Body weight was also measured 2.5 years after the intervention. DXA was not performed in the control group.

## Measurements

### Anthropometry

Body weight, waist circumference, hip circumference and sagittal abdominal diameter were measured in the fasting state at baseline, after the intervention and after 6 and 12 months. Two and a half years after the intervention, 31 (3) months in intervention group, 29 (5) months in control group, measurement of body weight was repeated either at our department or on calibrated scales elsewhere and self-reported by the subjects.

### Basal metabolic rate

Basal metabolic rate (BMR) (kcal/24 h) was measured by a ventilated hood technique (Delta Trac, Sensor Medics, Yorba Linda, CA, USA) at baseline, after the intervention and after 6 and 12 months. The duration of the registration of BMR continued for 15 minutes and a mean value of the last six 1-minute-based recording was calculated.

### Body composition

Body composition was measured with DXA (Dual energy X-ray Absorptiometry, Lunar Prodigy, GE Medical Systems, Diegem, Belgium) at baseline, after the intervention and when followed-up 12 months after the intervention. Bone mineral content (BMC) refers to total weight of skeleton and fat free mass (FFM) refers to total weight of non-bone and non-fat mass. Fat mass (FM) refers to weight (kg) fat mass of the total body weight without BMC and FFM and body fat (BF) refers to percentage fat tissue of total bodyweight without BMC. The trunk was separated from the arms and legs by a line passing the humeral head and the apex of the axilla. Android fat mass was determined as the area above the iliac crest defined as 20% of the distance from iliac crest to the base of skull (H) and gynoid fat mass was defined as 2.0 the size of the android area located 1.5 *H below the base of android region (hips).

### Laboratory measurements

Blood samples as shown in table three were drawn in fasting state at baseline, after the intervention and when followed-up 6 and 12 months after the intervention and analysed at the hospitals local laboratory as described elsewhere [20,21].

### Statistical analysis

Statistical analysis was made using the Statistical Package for the Social Sciences (SPSS version 15.0-18, Inc, Chicago, IL, USA). ANOVA and Student's paired and unpaired t-test were used within and between groups for comparison of anthropometrics, body composition and laboratory measurements. Means and SD are given. Linear correlations, Pearson correlation coefficient, were calculated. P-value <0.05 was considered to be statistically significant.

### Ethics

Regional Ethical Review Board Linköping, Sweden gave ethical approval and the study was carried out according to the declaration of Helsinki.

## Results

### Anthropometry and basal metabolic rate

**Figure 1:**
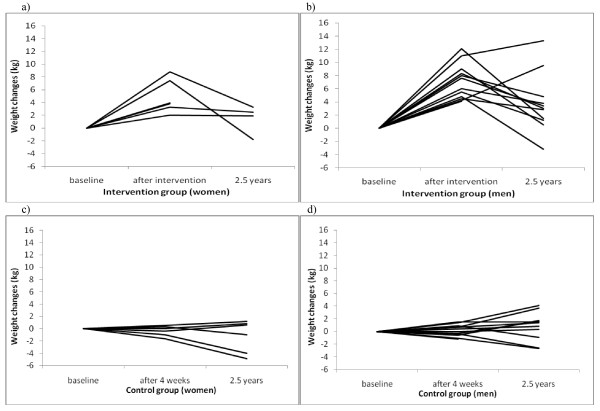
**Individual weight changes up to 2.5 years after hyper-alimentation in the intervention- and control group**. Individual weight changes (kg) in 18 healthy individuals before and after hyper-alimentation while simultaneously having a sedentary lifestyle for four weeks and when followed-up 2.5 years later compared with the control group of 18 individuals. Two and a half years after the intervention body weight showed a further increase (p = 0.01) in the intervention group while the weight of the controls was unchanged compared with baseline (p = 0.88). Changes in weight after 2.5 years significantly differed between the two groups (p = 0.015). The figures in the graph correspond to 6 women (a) and 12 men (b) in the intervention group and in 6 women (c) and 12 men (d) in the control group at baseline and after the intervention. Two and a half years after the intervention data was missing in 3 subjects of the intervention group and in one subject of the control group.

Changes in body weight and other anthropometric variables during the four week intervention are described in table 1. During the intervention there was an increase of body weight with 6.4 (2.8) kg and when followed-up 6 months after the intervention the subjects had lost 71 (50)% of this weight gain but they still had 1.6 (2.4) kg higher body weight than at baseline (p = 0.02). Five subjects had returned to a body weight of maximum +0.5 kg above their baseline body weight after 6 months as had 6 subjects after 12 months. Twelve months after the intervention the increase of body weight compared to baseline was 1.5 (2.4) kg (p = 0.018). Two and a half years after the intervention body weight showed a further increase by 1.3 (4.1) kg to on average 72.9 (8.9) kg in the intervention group. In the controls body weight was unchanged after 2.5 years compared to baseline, + 0.1 (2.5) kg (NS) which was significantly different from the increase found in the intervention group (p = 0.015). Individual weight changes for women and men in the intervention and control groups are shown in Fig 1a-d. At follow-up after 2.5 years an increase more than 5 kg was found in 2 subjects in the intervention group. BMR increased during the intervention but was unchanged compared to baseline values when followed-up 6 and 12 months after the intervention (Table 1).

**Table 1:** Anthropometrics in 18 normal weight individuals before and after an intervention with hyper alimentation while simultaneously having a sedentary lifestyle for four weeks and when followed-up 6 and 12 months after the intervention

	Baseline n = 18	After Intervention n = 18	6 months after intervention n = 16	12 months after the intervention n = 17	About 2.5 years after intervention n = 15	Baseline vs after intervention p-value	Baseline vs after 6 months p-value	Baseline vs after 12 months p-value	Baseline vs after 2.5 years p-value
Body weight (kg)									

*All*	67.6(9.1)	74.0(10.5)	68.6(9.3)	69.7(8.8)	72.9(8.9)	**<0.001**	**0.02**	**0.018**	**0.01**
*Women*	60(6.3)	64.8(8.1)	61.8(5.4)	61.7(7)	63.4(4.9)	**0.006**	**0.031**	0.22	0.28
*Men*	71.4(8)	78.5(8.5)	73.2(8.6)	74.6(6.5)	76.3(7.3)	**<0.001**	0.17	0.052	**0.02**

Waist circumference (cm)									

*All*	76.4(6.4)	83.1(7.9)	75.1(6.2)	76(5.4)	-	**<0.001**	0.71	0.46	-
*Women*	72.8(3.4)	79.2(7.2)	71.6(3.4)	73(3.2)	-	**0.022**	0.37	0.9	-
*Men*	78.1(6.9)	85.1(7.8)	78(6.8)	77.7(5.7)	-	**0.001**	0.86	0.33	-

Hip circumference (cm)									

*All*	86.4(7.1)	90.4(8.5)	83.3(4.7)	85.4(4.7)	-	**0.028**	0.2	0.15	-
*Women*	84.7(3.2)	90(8.4)	82.4(4.2)	84.2(3.9)	-	0.094	0.26	0.8	-
*Men*	87.4(8.4)	90.6(8.9)	84.1(5.4)	86.1(5.3)	-	**0.16**	0.36	0.14	-

Saggital abdominal diameter (cm)									

*All*	18.4(1.7)	20.4(1.6)	17.8(0.9)	18.4(1.6)	-	**<0.001**	0.83	0.81	-
*Women*	18(1.6)	19.5(1.9)	17.6(0.9)	17.6(0.9)	-	**0.048**	0.78	0.52	-
*Men*	18.6(1.8)	20.9(1.3)	18(0.9)	18.8(1.7)	-	**<0.001**	0.95	0.83	-

BMI (kg/m^2^)									

*All*	21.9(1.9)	23.9(2.2)	22.7(1.9)	22.5(1.9)	23.1(2.5)	**<0.001**	**0.017**	**0.028**	**0.012**
*Women*	22.0(2.0)	23.8(2.4)	22.7(2.0)	22.7(2.2)	22.7(2.0)	**0.005**	**0.032**	0.25	0.26
*Men*	21.8(2.0)	24(2.2)	22.6(1.9)	22.4(1.9)	23.2(2.7)	**<0.001**	0.18	0.062	**0.027**

Basal metabolic rate (kcal/24 h)									

*All*	1615(276)	1813(327)	1626(257)	1659(270)	-	**0.001**	0.48	0.32	-
*Women*	1326(128)	1483(132)	1393(180)	1412(203)	-	**0.036**	0.21	0.19	-
*Men*	1759(205)	1978(262)	1767(181)	1794(198)	-	**0.006**	0.98	0.77	-

Body weight in the controls (kg)	n = 18	n = 18			n = 17				

*All*	69.7(8.4)	69.7(8.7)	-	-	70.4(8.8)	0.96	-	-	0.88
*Women*	65.7(7.7)	65.4(7.5)	-	-	64.5(7.1)	0.32	-	-	0.31
*Men*	71.7(8.4)	71.8(8.7)	-	-	73.6(8.1)	0.54	-	-	0.25

All figures are means (SD). Body weight in the control group (n = 18) at baseline, after 4 weeks and after 2.5 years (lower part of table).

### Body composition

Fat mass increased from 12.7 (5.7) kg, corresponding to 20.1(9.8)% of total body weight at baseline to 16.4 (5.5) kg, corresponding to 23.8 (8.6)% after the intervention and almost half of this increase was present when followed-up 12 months after the intervention (Table 2) corresponding to an increase of 15.5 (16.2)% fat mass when followed-up at 12 months compared to baseline. During the intervention there was also a transient increase of fat free mass but when followed-up after 12 months there was no change compared to baseline values.

**Table 2:** Body composition in 18 healthy individuals as measured with Dual energy x-ray absorptiometry (DXA) before and after an intervention with hyper-alimentation while simultaneously having a sedentary lifestyle for four weeks and when followed-up 12 months later

	Baseline Women n = 6 Men n = 12	After intervention Women n = 6 Men n = 12	12 months after intervention Women n = 6 Men n = 10	Baseline vs after intervention* p-value	Baseline vs after 12 months* p-value
BF (%)					

*All*	20.1(9.8)	23.8(8.3)	22.6(8.9)	**<0.001**	**0.023**
*Women*	31.3(6.7)	33.4(6.4)	32.5(4)	0.08	0.45
*Men*	14.6(5.2)	19(4.4)	16.6(4.4)	**<0.001**	**0.011**

FM (kg)					

*All*	12.7(5.7)	16.4(5.5)	14.7(5.1)	**<0.001**	**0.01**
*Women*	17.9(4.2)	20.7(5.8)	19.3(3.5)	**0.037**	0.27
*Men*	10.1(4.4)	14.2(4)	11.9(3.7)	**<0.001**	**0.012**

FFM (kg)					

*All*	54.9(11.3)	56.7(11.5)	54.9(11.1)	**<0.001**	0.97
*Women*	41.9(5.3)	43.1(4.3)	42.4(4.6)	0.13	0.37
*Men*	61.5(6.8)	63.6(6.5)	62.4(5.3)	**<0.001**	0.54

Trunk fat (kg)					

*All*	6.5(2.9)	8.6(2.7)	7.5(2.5)	**<0.001**	**0.014**
*Women*	8.3(2.3)	9.6(3.1)	9.0(1.8)	0.097	0.29
*Men*	5.5(2.8)	8.1(2.5)	6.5(2.4)	**<0.001**	**0.022**

Gynoid fat mass (kg)					

*All*	2.8(1.2)	3.4(1.2)	3.2(1.1)	**<0.001**	**0.005**
*Women*	4.1(0.9)	4.5(1.1)	4.3(0.8)	0.038	0.24
*Men*	2.1(0.7)	2.8(0.6)	2.9(0.6)	**<0.001**	**0.005**

Android fat mass (kg)					

*All*	1.0(0.5)	1.4(0.5)	1.2(0.5)	**<0.001**	**0.032**
*Women*	1.3(0.4)	1.5(0.6)	1.4(0.3)	0.075	0.36
*Men*	0.9(0.6)	1.4(0.5)	1.0(0.5)	**<0.001**	**0.064**

BMC (kg)					

*All*	3.00(0.45)	3.03(0.46)	2.98(0.47)	**0.045**	0.69
*Women*	2.50(0.24)	2.52(0.28)	2.50(0.32)	0.61	0.9
*Men*	3.25(0.27)	3.29(0.28)	3.27(0.27)	**0.012**	**0.37**

FMI (kg/m^2^)					

*All*	4.3(2.2)	5.4(2.2)	4.9(2.1)	**<0.001**	**0.018**
*Women*	6.6(1.6)	7.6(2.1)	7.1(1.2)	**0.034**	0.31
*Men*	3.1(1.2)	4.3(1.2)	3.6(1.1)	**<0.001**	**0.011**

FFMI (kg/m^2^)					

*All*	17.6(2.4)	18.2(2.4)	17.6(2.3)	**<0.001**	0,80
*Women*	15.4(1.7)	15.8(1.2)	15.6(1.6)	0.15	0.33
*Men*	18.8(1.9)	19.4(1.8)	18.8(1.7)	**<0.001**	0.56

All figures are means (SD). All subjects participated at baseline and after the intervention. Twelve months after the intervention 16 subjects were included.

BF = Body fat, FM = Fat mass, FFM = Fat free mass, BMC = Bone mineral content, FMI = Fat mass index and FFMI = Fat free mass index.

*Comparisons are made with Students paired T-test.

Trunk fat mass increased by 2.2 (1.3) kg during the intervention, and when followed-up after 12 months the subjects had 0.75 (1.1) kg more trunk fat mass compared to baseline, corresponding to an increase of 17.5 (18.8)%. During the intervention android fat mass increased proportionally more than gynoid fat mass (android fat mass + 56.8 (54.1)%, gynoid fat mass + 28.9 (23.6)%, p = 0.003 between percentage increase of android respectively gynoid fat mass). Twelve months after the intervention only a tendency towards relative difference was found, the increase of android fat mass was 20.4 (23.6)% and gynoid fat mass 13.8 (13.3)% (p = 0.11 between percentage increase of android respectively gynoid fat mass).

Leg fat mass increased by 28.3 (23.6)%, (p < 0.001) during the intervention and when followed-up after twelve months fat mass in that area had increased by 14.5 (14.6)%, (p = 0.005) compared to baseline values. At 12 months there was about 15% increase of fat mass in the arms compared to baseline (NS).

There were no significant associations between increase of body weight during the intervention and increase of body weight after 31 months (r = 0.21, p = 0.44). In addition there was no correlation between fat mass at baseline and weight change during the intervention (r = -0.23, p = 0.36) or weight change after 12 months (r = -0.12, p = 0.68). There was a negative correlation between fat mass at baseline and change in fat mass after 12 months (r = -0.51, p = 0.046). BMR (kcal/24 h) increased during the intervention and was positively correlated to fat free mass derived from DXA, at baseline (r = 0.90, p < 0.001), after intervention (r = 0.92, p < 0.001) and when followed-up 12 months after the intervention (r = 0.87, p < 0.001).

### Laboratory measurements

At 12 months there was an increase of total cholesterol explained by increase of LDL-cholesterol while an increase of total triglycerides found at 6 months had returned to baseline levels at 12 months (Table 3). While there was no significant change of HDL-cholesterol concentration a decrease of apolipoprotein A1 was found at both 6 and 12 months (p < 0.01). A small non-significant increase of fasting insulin concentrations was found and there was no change in fasting plasma glucose values when followed-up both after 6 and 12 months. In HOMA-IR no statistical significant changes were found either at 6 or 12 months after the intervention. Liver transaminases were normal at the 12 month follow-up (Table 3).

**Table 3:** Lipoproteins, liver transaminases, glucose and insulin concentration in 18 normal weight subjects when followed-up 6 and 12 months after individuals participated in a four week intervention with hyper-alimentation while simultaneously having a sedentary lifestyle.

All Participants					
**Variable**	**Baseline n = 18**	**6 months n = 16**	**12 months n = 17**	**Baseline vs after 6 months p-value**	**Baseline vs after 12 months p-value**

Total cholesterol (mmol/l)	4.11(0.62)	4.16(0.68)	4.32(0.67)	0.17	**0.036**
Total triglycerides (mmol/l)	0.72(0.21)	0.91(0.30)	0.72(0.22)	**0.015**	0.98
LDL-cholesterol (mmol/l)	2.29(0.54)	2.29(0.63)	2.55(0.67)	0.28	**0.006**
HDL-cholesterol (mmol/l)	1.51(0.41)	1.46(0.46)	1.43(0.46)	0.25	0.22
Apolipoprotein A1 (g/L)	1.55(0.40)	1.26(0.31)	1.24(0.27)	**0.004**	**0.003**
Apolipoprotein B (g/L)	0.73(0.16)	0.79(0.18)	0.80(0.24)	0.86	0.49
ALP (μkat/l)	0.97(0.50)	1.16(0.86)	1.04(0.82)	0.12	0.57
ASAT (μkat/l)	0.48(0.21)	0.43(0.14)	0.45(0.25)	0.31	0.64
ALAT (μkat/l)	0.37(0.20)	0.43()0.37	0.42(0.36)	0.34	0.48
Fasting plasma glucose (mmol/l)	4.7(0.35)	4.8(0.29)	4.8(0.37)	0.45	0.75
Fasting insulin (pmol/L)	29.9(13.8)	37.9(27)	35(16.5)	0.16	0.23
HOMA-IR	0.9(0.4)	1.2(0.8)	1.1(0.5)	0.15	0.39

All figures are means (SD).

## Discussion

During the short-term intervention of four weeks, there was a marked increase of both fat free mass and of fat mass in both men and women [20,21]. Body weight increased by 6.4 kg. After the intervention the subjects could go back to their usual eating- and physical activity habits and when followed-up 6 months later their body weight had decreased, however not to baseline values. There were individual differences of weight changes after the intervention but only one third of the participants returned to +0.5 kg or less of their baseline body weight at 6 or 12 months after the intervention and in average there was an increase of body weight of 1.5 kg one year after the intervention period. Our hypothesis was that body weight should return to baseline after one year and we therefore did not measure body weight of the controls neither at 6 nor 12 months after their last visit. However, body weight was measured in both groups after 2.5 years and at that time there was a further increase in the subjects who participated in the intervention but body weight was unchanged compared to baseline in controls.

Siervo et al [16] overfed 6 subjects stepwise during 3 periods of 3 weeks each (+20, +40, +60% increase of their baseline energy intake) and found an increase of fat mass by 3.1 kg and of fat free mass by 2.7 kg during the last step. For comparison our subjects increased their energy intake with in average 70% during the whole intervention [20] and their increase of fat mass was 3.7 kg. Norgan & Durnin [18] overfed 6 healthy men (+1500 kcal/day) during 42 days and they found an average weight gain of 6 kg corresponding to a 10% weight gain, and the increase of fat mass was 3.7 kg. Both these studies included few participants but show similar changes in body composition as our study.

In our study body composition was followed-up 12 months after the intervention and at that time we found an increase of fat mass compared to baseline while the initial increase of fat free mass had returned to baseline levels. Previously, overeating experiments have been performed to describe effects of increased caloric intake in human beings [15-17] but only few long-term follow-up studies have been made [18,19]. The increase of fat mass, on average 1.4 kg, shows that the increase of body weight found after 12 months was fully caused by an increase of fat mass. In a study 23 young men were overfed during 100 days and followed-up 4 months later [17]. During the 100 days of overfeeding body weight increased with on average 8.1 kg; both fat mass and fat free mass increased. When those men were followed-up 4 months later they had, just like our subjects after 6 months, lost most of the body weight gain but not to origin. Effects of massive overfeeding by carbohydrates during 4-6 months, the Guru Walla session (a traditional fattening session in Cameroun), were described by Pasquet and Apfelbaum [22]. There was an increase of body weight by 19 (3.2) kg and of fat mass by 11.8 (2.5). After 30 months body weight had returned to baseline and only 5% of the increase of body fat remained but 1 participant who kept much of the gained body weight was excluded from the analysis.

Our finding of an increased body weight of 1.5 kg and fat mass of 1.4 kg over just 12 months raises the question whether a short period of overeating can induce a subsequent increase of fat mass. The suggestion of such an effect is supported by the fact that we did not observe a weight gain in the controls when body weight was measured after 2.5 years. Increases in body weight in adult individuals are common with increasing age but the changes described are smaller than what we observed. In Sweden a 10-year increase of body weight of 3.8 (6.0) kg has been found in individuals aged 25-64 years [6] which is clearly less than the increase we found in just one year if evenly distributed during the 10-year period. In the US obesity is more prevalent than in Sweden and increases of approximately 9.1 kg between the ages of 25-55 years have been described [2] and for most young adults there is a yearly increase of body weight of 0.2-0.8 kg [8]. The subjects in the Canadian study by Tremblay et al [17] were re-investigated 5 years later [19] and were found to have increased their body weight by 5 kg. Although this was a very large increase of body weight, it was concluded that there was no persistent effects of exposure to the overfeeding protocol over the expected age-associated increases in body mass, body fat, upper-body fat, abdominal visceral fat, and metabolic variables.

In a Swedish study lean healthy young adults free from health problems have been found to be the most likely group to gain weight [23] which might indicate that young individuals being overweight or obese have increased the awareness of obesity related problems and avoiding further weight gain. We lack knowledge of our participants' attitudes towards a healthy lifestyle, physical activity and eating habits before inclusion but on the other hand, most of them were medical students which is why we believe that they were aware of the potential risks by gaining body weight. The intervention was also clearly described to all participants. However, due to the demanding design we did not randomize subjects to the intervention or the control group, which might have biased the long-term results. Speculatively some of the subjects in the intervention group could be less cautious about gaining body weight as they volunteered to participate in the intervention.

The accumulation of trunk fat mass has been reported to be related to total body fatness in both genders [24] and in healthy non-obese men trunk fat mass measured with DXA has been found to have high correlation to visceral adipose tissue measured with MRI [25]. In the overfeeding study by Siervo et al [16] an increase of trunk fat mass was found and when fat volume derived from DXA was compared with abdominal fat volume derived from MRI they found visceral fat mass to have increased in greater extent than subcutaneous fat mass. We have previously reported an increase of abdominal fat volumes derived from MRI during the intervention [21] and men were more likely to accumulate fat mass in the abdominal region as visceral fat mass than women. In this study we found a non-significant tendency towards a greater increase of android than gynoid fat mass at 12 months. In addition the change appeared to be greater in men than in women but this was not statistically significant. We did not perform MRI 12 months after the intervention and cannot discern subcutaneous fat mass but the changes of trunk fat mass determined by DXA show that fat mass had increased above baseline values and fat free mass decreased to baseline in that area.

Weight gain due to overeating has been reported to be less than theoretically expected due to an increase of BMR [15]. The increase of BMR has however been found to be related to body weight [15,16,18]. We found an increase of BMR during the intervention [20,21] which secured our subject from an even greater weight gain but at the follow-up after the intervention BMR had returned to baseline in agreement with reduction of fat free mass to baseline level. Strong associations between fat free mass and BMR were found on all occasions confirming that fat free mass is of importance for BMR and can by itself reduce the risk for gaining extra body weight.

Interestingly we also found a small but significant increase of BMC during the intervention, which could be seen as an effect of increased body weight on bone. Measurements of BMC may on the other hand not completely correct for body size, especially when body size is changing dramatically [26] as in our study.

We have previously described an increase of serum ALT associated with increase of body weight and intake of energy from carbohydrates during the intervention [20]. This long-term follow-up shows no remaining effect on these liver enzymes in spite of the increase of fat mass found one year after the intervention. On the other hand a small deterioration of the lipid profile was found.

## Conclusion

In conclusion, excessive hyper-alimentation and limited physical activity changes body composition with an increase of both fat mass and fat free mass. The sustained increase of fat mass can be interpreted as a common change in this age group but it was larger than expected from epidemiological studies and also the clear difference between the body weight development in the intervention group and in controls raises the question whether there is remaining effect on fat mass after a short period of hyper-alimentation.

## Competing interests

The authors declare that they have no competing interests.

## Authors' contributions

FN conceptualized the study idea. ÅE, TL and, FN participated in the data analysis and contributed in writing the manuscript. All authors have read and approved the final manuscript.
